# Complete-Proteome Mapping of Human Influenza A Adaptive Mutations: Implications for Human Transmissibility of Zoonotic Strains

**DOI:** 10.1371/journal.pone.0009025

**Published:** 2010-02-03

**Authors:** Olivo Miotto, A. T. Heiny, Randy Albrecht, Adolfo García-Sastre, Tin Wee Tan, J. Thomas August, Vladimir Brusic

**Affiliations:** 1 Centre for Genomics and Global Health, University of Oxford, Oxford, United Kingdom; 2 Mahidol-Oxford Research Unit, Faculty of Tropical Medicine, Mahidol University, Rajthevee, Bangkok, Thailand; 3 Department of Biochemistry, Yong Loo Lin School of Medicine, National University of Singapore, Singapore, Singapore; 4 Department of Microbiology, Mount Sinai School of Medicine, New York, New York, United States of America; 5 Division of Infectious Diseases, Department of Medicine, Mount Sinai School of Medicine, New York, New York, United States of America; 6 Emerging Pathogens Institute, Mount Sinai School of Medicine, New York, New York, United States of America; 7 Department of Pharmacology and Molecular Sciences, Johns Hopkins University School of Medicine, Baltimore, Maryland, United States of America; 8 Cancer Vaccine Center, Dana-Farber Cancer Institute, Boston, Massachusetts, United States of America; Providence Health Care, Canada

## Abstract

**Background:**

There is widespread concern that H5N1 avian influenza A viruses will emerge as a pandemic threat, if they become capable of human-to-human (H2H) transmission. Avian strains lack this capability, which suggests that it requires important adaptive mutations. We performed a large-scale comparative analysis of proteins from avian and human strains, to produce a catalogue of mutations associated with H2H transmissibility, and to detect their presence in avian isolates.

**Methodology/Principal Findings:**

We constructed a dataset of influenza A protein sequences from 92,343 public database records. Human and avian sequence subsets were compared, using a method based on *mutual information*, to identify *characteristic sites* where human isolates present conserved mutations. The resulting catalogue comprises 68 characteristic sites in eight internal proteins. Subtype variability prevented the identification of adaptive mutations in the hemagglutinin and neuraminidase proteins. The high number of sites in the ribonucleoprotein complex suggests interdependence between mutations in multiple proteins. Characteristic sites are often clustered within known functional regions, suggesting their functional roles in cellular processes. By isolating and concatenating characteristic site residues, we defined *adaptation signatures*, which summarize the adaptive potential of specific isolates. Most adaptive mutations emerged within three decades after the 1918 pandemic, and have remained remarkably stable thereafter. Two lineages with stable internal protein constellations have circulated among humans without reassorting. On the contrary, H5N1 avian and swine viruses reassort frequently, causing both gains and losses of adaptive mutations.

**Conclusions:**

Human host adaptation appears to be complex and systemic, involving nearly all influenza proteins. Adaptation signatures suggest that the ability of H5N1 strains to infect humans is related to the presence of an unusually high number of adaptive mutations. However, these mutations appear unstable, suggesting low pandemic potential of H5N1 in its current form. In addition, adaptation signatures indicate that pandemic H1N1/09 strain possesses multiple human-transmissibility mutations, though not an unusually high number with respect to swine strains that infected humans in the past. Adaptation signatures provide a novel tool for identifying zoonotic strains with the potential to infect humans.

## Introduction

Influenza A is a virus belonging to the *Orthomyxoviridae* family, which circulates amongst various animal species. Although aquatic wildfowl are the natural reservoir, influenza A viruses routinely infect many types of domestic birds and several mammalian species. In humans, influenza A viruses are the cause of widespread annual epidemics and of less frequent pandemics, four of which were recorded within the last century [1]. The threat of a new pandemic is cause of the greatest concern, because of the prospect of high death tolls: the Spanish flu in 1918/19 claimed over 40 million lives, making it possibly the most destructive event in medical history [2]. The rapid spread and large-scale effect of pandemics are enabled by the presence of novel surface glycoproteins, hemagglutinin (HA) or neuraminidase (NA), for which the human population has no immune memory. Sixteen serologically distinct HA types, and nine NA types, are known to circulate in the avian host population; over 100 avian influenza subtypes have been catalogued to date, resulting from the combination of different HA and NA types. Only three of these subtypes, (H1N1, H2N2 and H3N2) are known to have circulated amongst humans in the last century. Other influenza subtypes of avian origin have infected humans without acquiring the ability to spread in the human population [3]. Current concerns focus on two pandemic threats: from the H5N1 highly pathogenic avian influenza (HPAI) and from the swine-origin H1N1/09 strain. H5N1 viruses have been responsible for a considerable number of human infections and deaths: according to the WHO, 395 individuals were infected by H5N1 between 2003 and 2008, resulting in 250 deaths (www.who.int/csr/disease/avian_influenza/). Although no definitive evidence of human-to-human (H2H) transmission of H5N1 has been reported, there is widespread concern that these viruses could cause a new devastating pandemic if they acquire such capabilities. The H1N1/09 swine strain was first reported to infect humans in April 2009 [4], and has been classified as pandemic by the WHO (http://www.who.int/csr/disease/swineflu/). Although this virus currently appears to cause mild disease [5], its rapid global spread is causing public alarm as the next epidemic season approaches. Despite the urgency, it is currently impossible to reliably predict the emergence of a new pandemic [1], and new tools are needed for scientists and policymakers to evaluate the pandemic risk posed by zoonotic viruses.

Limited spread of zoonotic influenza in humans indicates that immunological naivety of the host population is not a sufficient condition for initiating a human pandemic, and additional adaptive mutations in the virus are required. Such mutations appear not to be limited to the HA and NA proteins, but are also distributed across its nine internal proteins [6] (for conciseness, we will refer to all proteins other than HA and NA as “internal”, although a small domain of the M2 protein is externally exposed). A full reconstruction of this complex landscape of adaptive mutations is needed for elucidation of biological mechanisms of viral adaptations to humans. Detailed knowledge of adaptive mutations may also reveal whether zoonotic strains have the potential for acquiring H2H transmissibility without needing to reassort with human strains. A cost effective approach to identifying mutations of critical importance for host range is to conduct comparative analyses of large groups of human and avian protein sequences, to identify candidate mutation sites that can subsequently be experimentally validated. At such *characteristic sites*, a residue that is highly conserved within the human group but rarely observed in the pool of avian strains (a *characteristic variant*) is likely to be associated with an important adaptive mutation, whose loss would affect the ability of viruses to propagate amongst human hosts. Studies based on visual inspection of small numbers of representative isolates found characteristic sites in matrix proteins [7] and polymerases [8], [9]. Large-scale computational methods have used statistical variability measures such as *information entropy* to identify characteristic sites for human transmissibility. An analysis of 401 full viral proteomes [10] identified characteristic sites by comparing entropy in the avian and human groups, which limited its applicability to positions that are highly conserved in both groups. Finkelstein *et al.* [11] employed statistical tests that compared residue frequencies, to construct a catalogue of 32 characteristic mutations in five influenza proteins from the analysis of more than 23,000 sequences.

This report describes a large-scale complete-proteome analysis of influenza A sequences: a form of genome-wide association analysis in which a statistical measure is applied to compare two alignments of sequences, characterized by phenotype (human-adapted vs. non-adapted). This method, which uses *mutual information* as the statistical measure, was previously applied successfully to the study of the PB2 polymerase [12]. The catalogue of characteristic sites identified by our analysis was then applied to derive *adaptation signatures* of viral proteomes, which summarize the residue profiles at all characteristic sites for any given isolate. By rendering these signatures graphically, we reconstructed the history of the emergence of adaptive mutations in human-infecting influenza A viruses. We also used signatures to analyze the presence of H2H adaptive mutations in avian and swine viruses, and discussed the implications on the pandemic potential of zoonotic influenza.

## Materials and Methods

### Data Collection and Preparation

We compiled a dataset of all available influenza A sequences (as of September 2006) from the NCBI GenBank and GenPept databases [13], including entries mirrored from UniProt [14]. A total of 92,343 records were retrieved from these databases, using taxonomy-based queries; entries from different databases that referred to the same sequences were subsequently merged. If sufficient information was available, sequences were annotated with descriptive metadata properties: isolate name, host organism, subtype, year of isolation, geographic origin, and protein name. The resulting dataset was verified by two independent curators, who discarded duplicates, laboratory strains, sequences with missing key metadata, and sequences with quality issues. The final set comprised a total of 40,169 unique sequences, including both full-length sequences and fragments, covering all influenza A proteins. The data collection and cleaning process was largely automated by the Aggregator of Biological Knowledge (ABK) tool, which uses a rule-based approach to aggregating data from multiple database sources [15].

For each of the eleven influenza proteins, a master multiple sequence alignment (MSA) was constructed using the MUSCLE 3.6 [16] software. The MSAs were manually inspected and corrected. Multiple subset alignments, to be used in comparative analyses, were extracted from the master alignments based on their metadata values, using the Antigenic Variability Analyzer (AVANA, http://avana.sourceforge.net), developed by the authors to support information-theoretical analysis tasks [17], [18]. AVANA was also used to conduct all comparative analyses described in this paper.

### Subset Selection

**Figure 1:**
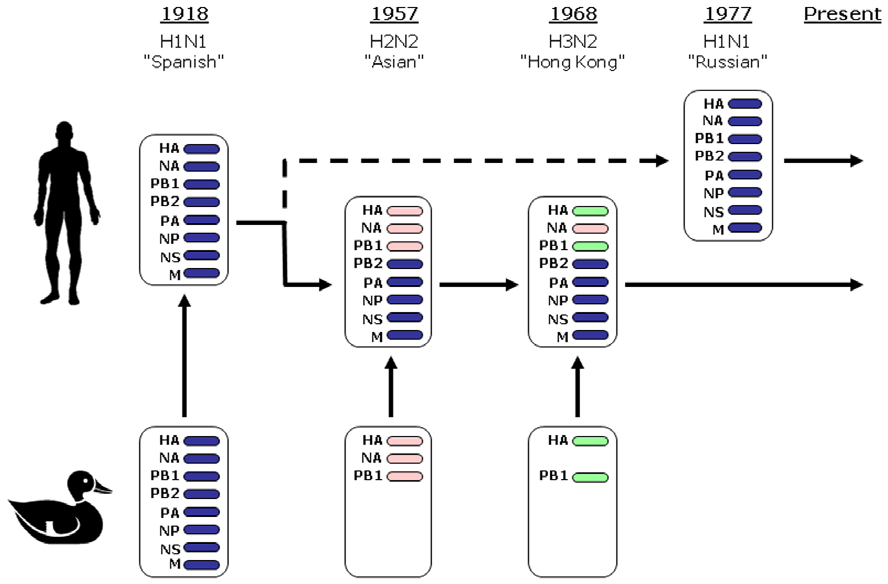
Human Influenza A reassortment events of the 20^th^ Century. The reassortment events associated with human pandemics in the 20^th^ century (adapted from Webster *et al.* [39]). A full complement of eight gene segments of zoonotic origin caused the 1918 Spanish flu. In 1957, the H2N2 Asian flu pandemic replaced the HA, NA and PB1 segments, and in 1968 the H3N2 Hong Kong pandemic replaced the HA and PB1 segments only [40]. In both cases, the new subtype fully replaced the subtypes previously circulating amongst humans. The 1977 Russian epidemic introduced an H1N1 strain almost identical to that circulating prior to 1957, and may have been caused by the release of 20-year old frozen viruses [19]. The H1N1 and HxN2 lineages have since co-circulated in the human population; recently, their reassortment has given rise to human strains of H1N2 subtype.

The objective of this study was to identify sites where characteristic mutations are present in the majority of human influenza A viruses. Two major lineages of human influenza A are currently co-circulating: H3N2 and H1N1. In spite of their common origin (Figure 1), the internal protein constellations of these two lineages have evolved independently, following the disappearance of H1N1 in 1957 and its reintroduction in 1977 [19]. Because of genetic similarity and common descent, the internal proteins of subtypes H2N2 and H1N2 were grouped with H3N2 in a lineage named HxN2, while H1N1 formed the other lineage. For each of the nine internal proteins, three subsets were therefore extracted: **A2A** (all avian sequences, except for H1N1, H2N2, H1N2, H3N2 and H5N1 subtypes), **H1N1H** (all H1N1 human sequences) and **HxN2H** (all human sequences of subtypes H2N2, H1N2 and H3N2). Since true adaptive mutations are expected to be present in both lineages, we analyzed each lineage separately, discarding sites that are not shared by both human influenza lineages. Although subtype H1N2 has not been confirmed to be a stable circulating human subtype, it was included in the HxN2H group because of the strong sequence similarity of its internal proteins to that group: we found that removing this subtype from the analysis does not change our catalogue of characteristic sites. H5N1 sequences were removed from both avian and human subsets because of this subtype's pronounced ability to jump the species barrier.

In a subsequent refinement of the NS1 analysis, we separated sequences for this protein into two groups, corresponding to the two major NS variants known as Alleles A and B, whose nucleotide sequences exhibit approximately 70% identity [20], [21]. Sequences were classified based on similarity to a reference Allele B isolate (A/pintail duck/ALB/121/1979 (H7N8)). While 31% of A2A sequences were classified as Allele B, all H2H sequences were found to belong to Allele A, supporting the hypothesis that human influenza A evolved from an avian Allele A lineage [22]. Comparisons restricted to Allele A sequences are therefore more likely to identify mutations caused by host adaptation rather than lineage difference. H2H sequences were compared against the Allele A subset of A2A sequences, and only characteristic sites that met our selection criteria in this comparison were selected for our final catalogue.

To analyze adaptation signatures for different virus groups, we also collected subsets of avian H5N1 (**H5N1A**) and human H5N1 (**H5N1H**) sequences, as well as subsets of swine (**SW**) and equine (**EQ**) sequences. The number of sequences in each of the extracted datasets is included in Table 1. Because of the high degree of genetic divergence in the glycoproteins, we compared separately each subtype that has circulated amongst humans: H1, H2 and H3 subtypes of the HA protein, and N1 and N2 subtypes of NA. Table 2 shows the number of sequences included in each HA subset, and Table 3 shows the subset sizes for the NA proteins.

**Table 1:** Count of influenza A internal protein sequences used in the current study.

	*Sequences used in MI Analysis*				*Additional sequences used in signature analysis*				
	A2A	H1N1H	HxN2H	Total	H5N1A	H5N1H	SW	EQ	Total
**M1**	1047	300	1521	**2868**	458	105	190	22	**775**
**M2**	736	286	1517	**2539**	289	95	104	21	**509**
**NP**	884	316	1645	**2845**	420	114	230	22	**786**
**NS1**	1123	303	1448	**2874**	457	95	172	38	**762**
**NS2**	810	292	1419	**2521**	288	81	113	21	**503**
**PA**	701	279	1362	**2342**	402	102	161	11	**676**
**PB1**	716	303	1385	**2404**	400	101	163	19	**683**
**PB2**	719	281	1369	**2369**	404	97	161	17	**679**
**PB1-F2**	352	262	1280	**1894**	-	-	-	-	-
**Total**	**7088**	**2622**	**12946**	**22656**	**3118**	**790**	**1294**	**171**	**5373**

Characteristic site analysis was conducted using the A2A, H1N1H and HxN2H sets. The H5N1A, H5N1H, SW and EQ sets were used for sequence signature analysis.

**Table 2:** Count of influenza A hemagglutinin protein sequences used in the current study.

	Avian	Human	Total
**H1**	48	768	**816**
**H2**	80	75	**155**
**H3**	115	3105	**3220**
**Total**	**243**	**3948**	**4191**

**Table 3:** Count of influenza A neuraminidase protein sequences used in the current study.

	Avian	Human	Total
**N1**	717	360	**1077**
**N2**	439	1801	**2240**
**Total**	**1156**	**2161**	**3317**

### Identification of Characteristic Sites and Variants

The method for identifying characteristic sites has previously been described in detail [12]. Briefly, an aligned set of *adapted* sequences (capable of H2H transmissibility) was compared against a *reference* set (not H2H-transmissible) to reveal mutations common in the adapted set, but rare in the reference set. To measure the strength of association between mutations and sequence sets, we used *mutual information* (MI), an information theoretical statistic that measures the strength of association between a pair of variables [23]. MI is defined in terms of *information entropy*, a measure of variability. The information entropy *H*(*x*) of a discrete variable *x* is given by:
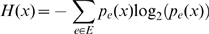
(1)where *E* = {*e_1_*, *e_2_* … *e_n_*} is the set of all possible discrete values of *x*, and *p_e_*(*x*) is the probability that *e*∈*E* is the value of *x*. The mutual information between two variables A and B is then defined by:
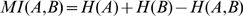
(2)where *H*(*A*) and *H*(*B*) are entropies of A and B, while *H*(*A*,*B*) is their *joint entropy*, computed from equation (1) by replacing *E* with the set of all unique pair of values (*A*,*B*).

To identify characteristic sites, we have modified equation (2) to compute the MI between an observed residue *a* at position *x*, and the label *S* of the set (alignment) within which residue *a* is observed:
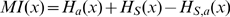
(3)
*H_a_*(*x*) is the entropy in an alignment formed by merging the two set, while *H_S_*(*x*) is derived from the number of sequences in each of the two sets (*n_1_* and *n_2_*):
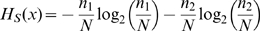
(4)where *N* = *n_1_* + *n_2_*. Finally, *H_S_*_,*a*_(*x*) is given by:
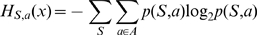
(5)where *p*(*S*,*a*) is the probability of any given combination of residue and set label.

When comparing two sets of equal size, the above equations give MI values in the range 0≤*MI*(*x*)≤1. At alignment sites with high MI, the observed residues are strongly associated to a given set; conversely, sites with low MI exhibit similar distributions of residues in the two sets.

As equation (4) suggests, MI is reduced when the two sets have unequal sizes. To compensate for this bias and standardize results, we have applied a statistical correction, based on repeated resampling. For each pair of sets, we repeatedly compared the smaller set to a set of equal size, randomly sampled without replacement from the larger set. The final MI values were the average over 1000 iterations. This method is effective in correcting the size bias [12], and confers robustness to the MI measurement. In addition, by selecting strains randomly across all phylogenetic groups, it partially corrects for phylogenetic sampling biases.

Characteristic sites and their characteristic variants (mutations) were selected based on four empirical criteria, whose rationale is detailed in [11], and summarized as follows:

- At a characteristic site, MI≥0.4 (found to be the MI threshold below which avian and human sequences converge to the same consensus amino acids).
- A characteristic variant must be 4 times more common in one set than in the other set (threshold determined from variant distribution analysis for the PB2 and NS1 proteins)
- A characteristic variant must occur in at least 2% of the sequences within the set it represents (a threshold found to be a good compromise between the minimum representation of characteristic mutations and the maximum representation of non-characteristic mutations in PB2)
- An avian characteristic variant must be uncommon in the H2H set at a characteristic site. We have manually inspected all sites where avian variants accounted for more than 2% of sequences in at least one H2H lineage. All accepted characteristic sites had less than 5.2% avian variants (average at all characteristic sites was 0.71%).

Only characteristic sites present in both H2H lineages were included in the final catalogue.

### Reconstruction of Adaptation Signatures

The variants that distinguish H2H sequences and A2A sequences at characteristic sites form a *characteristic variant pattern*, a summary of the significant differences between the two sets of sequences across the whole proteome. This pattern was used to construct the *adaptation signatures* of several influenza proteomes, by discarding all residues except those at characteristic sites. Residues forming the signatures were tagged as A2A-like (*i.e.* a characteristic variant of the A2A subset), H2H-like (an H2H characteristic variant), or as non-characteristic. The resulting signatures thus provide a succinct summary of H2H adaptive mutations contained in any influenza proteome. To facilitate the evaluation of multiple isolates, we developed a software program to graphically display selected signatures along a timeline, using a contrasting color scheme to distinguish between A2A-like and H2H-like residues.

## Results

### Catalogue of Characteristic Sites

**Figure 2:**
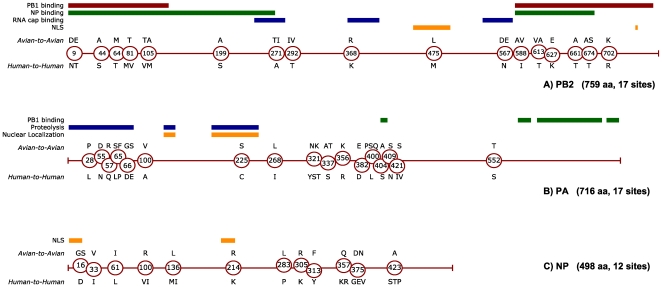
Characteristic sites identified in components of the RNP assembly of influenza A (PB2, PA, NP proteins). Circular markers, indicating the position of characteristic sites, are placed along the sequence length of the PB2 (A), PA (B) and NP (C) proteins of influenza A. Avian (A2A) variants are indicated above each marker, while human (H2H) variants are located below. If multiple characteristic variants are present, they are shown in decreasing order of frequency. In the upper part of each figure, colored lines show reported functional domains of PB2 [41]–[44], PA [45]–[48] and NP [49].

**Figure 3:**
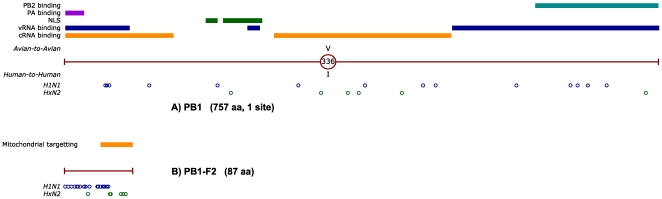
Characteristic sites identified in the PB1 (A) and PB1-F2 (B) proteins of influenza A. RNA segment 2, which encodes both the PB1 and PB1-F2 proteins, has been replaced at the onset of the 1957 and 1968 pandemics (see Figure 1). As a result, the H1N1 and HxN2 lineages do not share recent common origin for this segment. Characteristic mutations are therefore shown separately for the two lineages, in the lower part of each diagram, using blue (H1N1) and green (HxN2) circles. Known functional sites for PB1 [46], [50]–[52] and PB1-F2 [53] are also indicated by colored lines in the upper part of each figure.

**Figure 4:**
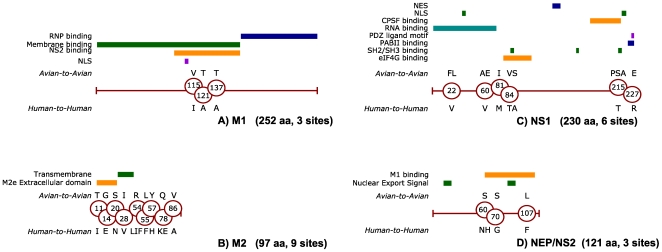
Characteristic sites identified in the matrix proteins M1 (A) and M2 (B) and non-structural proteins NS1 (C) and NEP/NS2 (D) of influenza A. Identified characteristic sites are mapped against known functional domains of M1 [54], [55], M2 [56], NS1 [57]–[64] and NEP/NS2 [65], [66], using the notation used in Figure 2.

**Figure 5:**
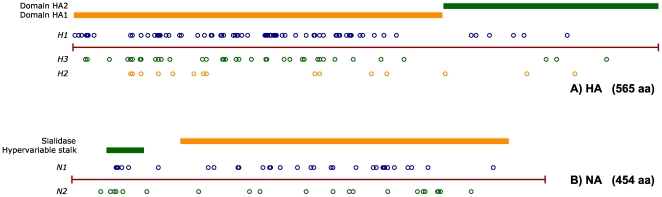
Characteristic sites identified in the HA (A) and NA (B) glycoproteins of influenza A. The characteristic mutations identified for each of the subtypes present in humans are shown: H1 (blue circles), H2 (green circles), H3 (orange circles) for HA; and N1 (blue circles), N2 (green circles) for NA (details of these sites are given in Tables S1, S2, S3, S4, S5 of the Supplementary Materials S1). Known domains of these two proteins are indicated by coloured lines in the upper part of each figure.

Our analysis produced a catalogue of 68 characteristic sites that met selection criteria (Table 4). Characteristic sites were found in eight of the nine internal proteins, suggesting that adaptation to humans requires participation of most products encoded by the viral genome. The location of characteristic sites found within the internal proteins is shown in Figures 2, 3 and 4, alongside the mapping of known functional domains in these proteins. As shown in Figure 2, the three internal proteins found to contain the highest number of characteristic sites were PB2 (17 sites), PA (17 sites), and NP (12 sites). These three proteins, responsible for the transcription and replication of viral RNA, bind to each other, to the PB1 polymerase and to viral RNA to form the ribonucleoprotein (RNP) complex that encases each of the 8 genomic segments packaged within the virion. However, the PB1 protein was found to contain only a single characteristic site. PB1 and PB1-F2 are encoded by an RNA segment that was replaced during the 1957 and 1968 pandemics (Figure 1). As a result, adaptive mutations found in these two proteins are lineage-specific, with one notable exception: a single PB1 site has independently produced the same adaptive mutation (V336I) in both H1N1 and HxN2 lineages (Figure 3). All remaining internal proteins were found to contain multiple characteristic sites: M1 (3 sites), M2 (9 sites), NS1 (6 sites) and NEP/NS2 (3 sites), as shown in Figure 4. The M2 protein contained the highest density of characteristic sites (almost 1 every 10 residues), including three sites within the M2 extracellular region (M2e), which has recently been proposed as a universal vaccine target [24]. The analysis of the HA and NA glycoproteins revealed a large number of subtype-specific adaptive mutations, as shown in Figure 5 (details are given in Tables S1, S2, S3, S4, S5 in the Supplementary Materials S1). However, we were unable to identify with confidence any adaptive mutation in these proteins as universal in all human-transmissible strains, even at those positions where mutations were found to occur in multiple subtypes.

**Table 4:** Full catalogue of identified characteristic sites for H2H transmission of influenza A.

Protein	Position	A2A		H2H			H1N1 CV	HxN2 CV
		CV	Cons	CV	Cons	X-pres		
M1	115	V	99.70%	I	99.39%	0.61%	I	I
	121	T	94.94%	A	99.89%	0.11%	A	A
	137	T	99.60%	A	99.23%	0.77%	A	A
M2	11	T	97.28%	I	96.89%	3.11%	I	I
	14	G	95.99%	E	98.28%	1.72%	E	E
	20	S	97.14%	N	97.94%	2.06%	N	N
	28	I	76.36%	V	97.72%	2.11%	V	V
	54	R	98.91%	LIF	98.94%	0.61%	IL	LF
	55	L	79.18%	F	99.33%	0.67%	F	F
	57	Y	99.59%	H	97.38%	2.18%	H	H
	78	Q	99.72%	KE	99.26%	0.28%	EK	K
	86	V	99.84%	A	99.21%	0.45%	A	A
NP	16	GS	99.16%	D	99.49%	0.51%	D	D
	33	V	99.76%	I	98.97%	1.03%	I	I
	61	I	98.36%	L	99.43%	0.57%	L	L
	100	R	99.65%	VI	99.71%	0.06%	V	VI
	136	L	85.41%	MI	99.77%	0.11%	I	MI
	214	R	96.64%	K	99.32%	0.68%	K	K
	283	L	100.00%	P	99.48%	0.47%	P	P
	305	R	99.17%	K	99.33%	0.67%	K	K
	313	F	99.31%	Y	99.48%	0.52%	Y	Y
	357	Q	98.43%	KR	99.90%	0.10%	KR	K
	375	DN	96.93%	GEV	99.34%	0.56%	V	GE
	423	A	97.06%	STP	98.88%	1.00%	T	SP
NS1	22	FL	97.07%	V	98.21%	0.40%	V	V
	60	AE	97.59%	V	99.20%	0.69%	V	V
	81	I	98.66%	M	99.08%	0.69%	M	M
	84	VS	96.08%	TA	99.20%	0.80%	A	TA
	215	PSA	99.24%	T	99.37%	0.63%	T	T
	227	E	98.87%	R	99.53%	0.06%	R	R
NS2	60	S	76.77%	NH	98.89%	0.82%	H	N
	70	S	97.46%	G	99.88%	0.12%	G	G
	107	L	99.60%	F	98.77%	1.17%	F	F
PA	28	P	100.00%	L	99.14%	0.67%	L	L
	55	D	99.69%	N	99.63%	0.37%	N	N
	57	R	96.61%	Q	98.72%	0.79%	Q	Q
	65	SF	99.08%	LP	99.63%	0.37%	PL	L
	66	GS	99.69%	DE	98.84%	1.10%	ED	D
	100	V	96.15%	A	99.27%	0.37%	A	A
	225	S	98.61%	C	99.39%	0.61%	C	C
	268	L	98.84%	I	99.14%	0.73%	I	I
	321	NK	97.35%	YST	97.30%	0.74%	STY	Y
	337	AT	99.34%	S	99.75%	0.25%	S	S
	356	K	98.51%	R	99.26%	0.74%	R	R
	382	E	94.34%	D	97.37%	2.45%	D	D
	400	PSQ	89.32%	L	99.45%	0.31%	L	L
	404	A	99.48%	S	99.39%	0.55%	S	S
	409	S	91.49%	N	99.45%	0.49%	N	N
	421	S	98.91%	IV	97.79%	0.55%	I	IV
	552	T	99.81%	S	99.75%	0.12%	S	S
PB1	336	V	96.66%	I	95.98%	4.02%	I	I
PB2	9	DE	98.57%	NT	99.33%	0.49%	N	NT
	44	A	96.82%	S	99.27%	0.61%	S	S
	64	M	97.29%	T	99.58%	0.30%	T	T
	81	T	97.93%	MV	99.27%	0.30%	VM	M
	105	TA	98.41%	VM	99.45%	0.36%	VM	VM
	199	A	99.47%	S	99.76%	0.24%	S	S
	271	TI	98.59%	A	99.51%	0.37%	A	A
	292	IV	95.54%	T	99.15%	0.67%	T	T
	368	R	98.12%	K	99.33%	0.67%	K	K
	475	L	99.66%	M	99.76%	0.24%	M	M
	567	DE	98.28%	N	99.39%	0.55%	N	N
	588	AV	98.45%	I	99.63%	0.31%	I	I
	613	VA	98.28%	T	96.82%	0.61%	TI	T
	627	E	99.31%	K	99.76%	0.12%	K	K
	661	A	86.72%	T	99.39%	0.43%	T	T
	674	AS	95.69%	T	99.63%	0.18%	T	T
	702	K	89.70%	R	99.39%	0.49%	R	R

The 68 characteristic sites identified by this study are shown in this table, grouped by protein. Each row represents a site, with the columns detailing the following: the protein name; the site position within the protein sequence; the A2A characteristic variant(s) and their conservation in the A2A subset; the H2H characteristic variant(s), their conservation in the H2H subset, and the contamination with avian variants observed in the H2H subset; the characteristic variant(s) observed in the H1N1 subset alone; and the characteristic variant(s) observed in the HxN2 subset alone.

Our catalogue of characteristic sites is considerably more extensive than those reported in related work. The most comprehensive previous study [11] used a large-scale dataset comparable in size to ours to identify 32 of the 68 characteristic sites found in the present work, indicating that MI may be a more sensitive measure of association than the statistical tests employed in that study. Chen *et al.* [10] identified 52 sites in ten proteins. Of these, 38 are present in our catalogue; our study discarded 12 sites shown to be representatives of a single lineage, and we were unable to identify two characteristic sites in HA reported by [10].

### Emergence of H2H Adaptive Mutations

**Figure 6:**
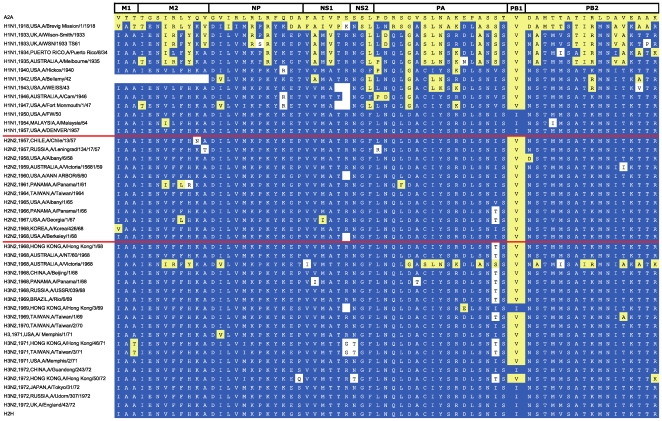
Timeline of adaptation to H2H transmission for the influenza A proteome. Adaptation signatures from human isolates between 1918 and 1972 are arranged in chronological order. Subtype, year and country of isolation, and isolate name are shown in the first column. The remaining columns show residues at all characteristic sites, in the order given in Table 4. A2A characteristic mutations are shown on a dark blue background, H2H mutations on a yellow background, while all other variants are on white. Blank cells represent unknown residues in incompletely sequenced proteomes. Consensus signatures for A2A and H2H proteomes are shown in the first and last row, respectively. Red horizontal lines indicate the start of the 1957 and 1968 pandemics, which introduced the H2N2 and H3N2 subtypes respectively.

To assess the stability of H2H characteristic mutations, and reconstruct the timeline of their emergence in human strains, we produced adaptation signatures for all available virus proteomes isolated from human hosts. Figure 6 shows the chronological display of signatures from viruses isolated between 1918 and 1972, a period spanning the three major 20^th^ Century pandemics. A2A and H2H characteristic residues are shown on contrasting backgrounds, making it easy to discern visually the evolutionary pattern of their emergence. The Spanish influenza pandemic isolate A/BrevigMission/1/1918 (H1N1), at the start of the timeline, is the oldest characterized proteome. Although this strain had a primarily avian signature, it contained 23 out of 68 H2H characteristic mutations (34%), distributed in all proteins except for PB1 and NS1. This number of H2H mutations is far higher than that of other avian strains in our dataset, all of which contain no more than eight H2H mutations. The 1918 H2H mutations were conserved in later human strains, which gradually accumulated additional adaptive changes throughout the 1930s and 1940s. By 1950, viruses with signatures with no avian characteristic variants were circulating, such as A/FW/50 (H1N1). Both the 1957 and 1968 pandemics (indicated by red lines) left the internal protein constellation practically unchanged, except for the replacement of the PB1 segment, which removed from circulation the V336I mutation developed in the 1950s by the H1N1 strains. However, this mutation re-emerged shortly after the 1968 pandemic: by 1972, the HxN2 lineage acquired full H2H signature. Five years later, a new pandemic introduced a human-adapted H1N1 strain, whose signature was identical to that of pre-1957 H1N1 strains (not shown in the figure), but different from that ofHxN2, which had diverged in the intervening years. Both lineages are still co-circulating today, and their signatures have remained distinct and stable throughout the intervening half-century. A comparison of all H1N1 and HxN2 signatures since 1977 revealed no indication of stable reassortments between the two lineages (data not shown). A2A mutations could only be found in isolates from reported infections of zoonotic origin, from swine (see A/Victoria/1968 in Figure 6) or avian hosts (for example, human H5N1 infections). Apart from major pandemics, we found no evidence that any zoonotic infection over the past 90 years has generated stable human-transmissible lineages.

### Assessment of Avian Strains for H2H Adaptive Mutations

**Figure 7:**
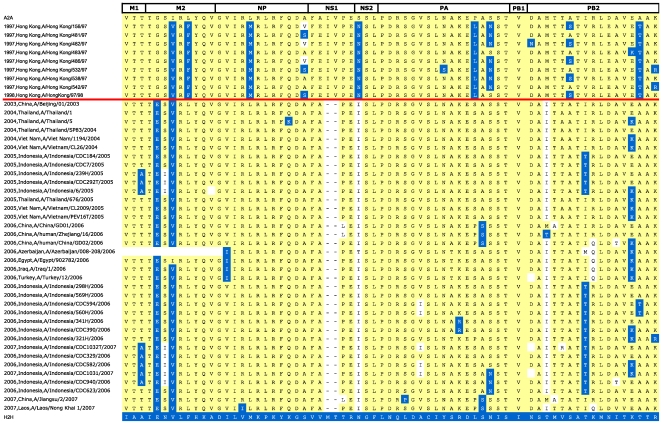
Adaptation signatures of human-isolated H5N1 influenza A proteomes. This figure shows the adaptation signatures of H5N1 sequences that infected humans in the period 1997–2008. For display clarity and conciseness, only a selection of representative signatures is presented. The same coloring scheme was used as in Figure 6. Dashes (in the NS1 protein signature) indicate amino acid deletions. A red horizontal line separates the early wave of infections in Hong Kong (1997–8) from more recent South-East Asian infections (since 2003).

We investigated the presence of adaptive mutations in avian strains by constructing adaptation signatures for all avian sequences analyzed in this study. The majority of avian signatures (>63%) contained no H2H mutations at all. Although this high percentage may be an overestimate (many of these signatures were incomplete due to partial sequencing of the source genomes), it is clear that H2H variants are rare in the avian influenza population. In contrast, we found an unusually high number of H2H mutation in human-infecting H5N1 strains, which are arranged chronologically in Figure 7, showing that two distinct signatures characterized two major “waves” of H5N1 infections. The 1997/98 Hong Kong isolates present up to ten H2H mutations spread over five internal proteins (*e.g.* A/Hong Kong/532/97 (H5N1)), more than any other avian strains in our dataset. Later strains, which spread to South-East Asia, Africa and Europe since 2003, also contain several H2H variants, but their number (between 3 and 6) is considerably lower than observed in the first wave. Only a single mutation was present in the majority of isolates in both waves: Ile→Val at position 28 in the transmembrane region of the M2 protein. In both waves, the signatures of human-infecting isolates were consistent with those of contemporary avian isolates in the same geographical region. Our study found several sequences with a high numbers of adaptive mutations from avian subtypes (see Figure S1 of the Supplementary Materials S1). Most of these viruses were isolated in Asia over the past decade, and belong predominantly to three subtypes (H5N1, H9N2 and H6N1). The presence of shared H2H mutations suggests that reassortments of multiple internal proteins have occurred between these three subtypes.

### Analysis of Signatures in Swine and Equine Isolates

**Figure 8:**
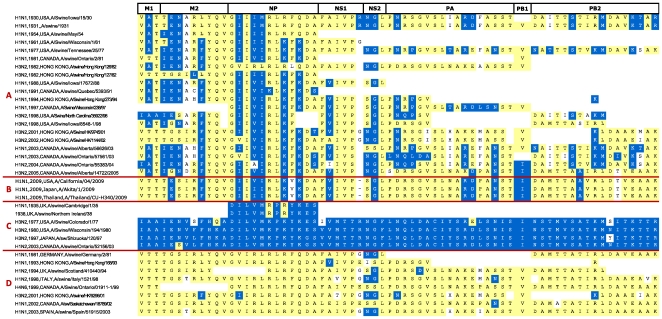
Adaptation signatures of selected swine influenza A proteomes. H2H signatures for a number of representative swine proteomes are shown. Subtype, year and country of isolation, and isolate name are shown in the first column, while the remaining columns show the signature residues, using the same coloring scheme as in Figure 6. The isolates are shown in four groups, according to signature similarity: (A) isolates with signatures similar to that of “classical swine” influenza, such as A/Swine/Iowa/15/30; (B) isolates from the H1N1/09 pandemic; (C) isolates with signatures that are very similar to those of contemporarily circulating human isolates; (D) isolates with predominantly “avian” signatures, containing very few or no H2H adaptive mutations. In most of groups, reassortment events are evidenced by discontinuities in the signature timeline.

We obtained adaptation signatures in our SW subsets (Figure 8), which clearly show that pigs are infected by a wide variety of influenza A viruses: in addition to signatures derived from early “classical swine” influenza (group A in Figure 8), we identified signatures typical of human (group C) and of avian viruses (group D). This data supports the hypothesis that swine hosts may be “mixing vessels” for the reassortments of avian and human influenza viruses, since they possess cell surface receptors that are bound by the HA of both avian and human influenza viruses [25]. Additionally, we found a small number of isolates with radically different signatures (see Figure S2 of the Supplementary Materials S1), consistent with the hypothesis that additional adapted lineages circulate among pigs [26]. The signatures of the pandemic H1N1/09 strains (group B in Figure 8) present strong similarities to previously circulating swine strains, although the signatures of three polymerase proteins are atypical, supporting the hypothesis of a recombinant virus [27]. However, similarities with the A/Swine/Albert/14722/2005 signature suggest that this recombinant virus has been in circulation for several years in the swine population. Further evidence of widespread reassortments is clearly visible in Group A signatures that acquired internal proteins possessing avian signatures. All internal proteins appear to be susceptible to such reassortments, for which we found no discernible pattern. Thirteen H2H adaptive mutations have been continually present in swine influenza strains over the last 70 years, suggesting they play an important adaptive role in swine-to-swine transmission. However, the absence of these mutations in many signatures suggests they are not a requirement for swine infection. Eleven of these conserved mutation were present in the 1918 Spanish influenza signature (M1 121; M2 14, 20; NP 33, 100, 136; NEP/NS2 60; PA 55; PB2 199, 475, 627), supporting the hypothesis of a common origin [28].

**Figure 9:**
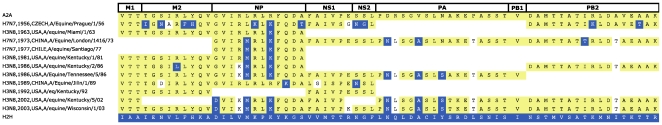
Adaptation signatures of selected equine influenza A proteomes. The signatures of selected equine isolates, spanning a period of nearly 50 years are shown. For conciseness, a number of similar signatures were removed from this set. Subtype, year and country of isolation, and isolate name are shown in the first column, while the remaining columns show the signature residues, using the same colouring scheme as in Figure 6.

A similar analysis of equine influenza signatures, conducted using the EQ dataset, revealed that they have predominantly avian signatures (Figure 9). Although the limited available data prevented us from making statistically significant observations, we note five H2H mutations (in the NP and PA proteins) that have appeared over several decades and may be conserved in circulating adapted strains. Of these mutations, only one (PA D55N) was also conserved in “classical swine” lineages as well as human lineages.

## Discussion

### Characteristic Sites Catalogue

The analysis described in this paper produced the most complete catalogue of H2H adaptive mutations published to date. This catalogue describes a complex landscape of adaptations, involving a greater number of proteins than reported by previous studies. The presence of characteristic sites in eight of the nine internal influenza proteins indicates that host adaptation is highly complex and systemic in nature, requiring the participation of products from the whole genomic ensemble. Gradual emergence of H2H mutations in the three decades after the Spanish influenza pandemic suggests that many of these adaptive mutations are individually not essential. However, their high level of conservation over the following decades strongly implies their important role in adapting to human hosts. A possible explanation is that the 1918 H1N1 proteome contained a non-optimal set of important components for human transmission, which has been refined over time to improve equilibrium between virus and host. This model does not imply that any of the 1918 mutations are individually sufficient, or necessary, for human-to-human transmission. There may be multiple combinations of H2H mutations capable of enabling sufficiently efficient infection and transmission in humans to allow the gradual refinement of the adaptive mutation repertoire. Our catalogue of characteristic sites, derived from the analysis of 90 years of refinements in human lineages, can therefore be a valuable tool for assessing the potential of zoonotic viruses to infect and circulate amongst humans.

Our results indicate that concurrent mutations in the internal protein constellation are required for efficient host range adaptation, although the role of most internal proteins is still poorly understood. Internal proteins participate in various cellular processes, such as nuclear transport, replication and virion assembly, each of which may require adaptation to the host organism. The location of characteristic sites within putative nuclear localization signals (NLS) of various components supports this model. However, it is unlikely that all characteristic sites identified in our catalogue play independent roles. The presence of multiple H2H characteristic sites in both M1 and NEP/NS2, within their reciprocal binding regions, raises the question of whether such mutations have co-evolved as a result of preferred structural interactions. This may also be the case for RNP complex proteins, which frequently contain characteristic sites in putative protein-binding domains. Using our previously published PB2 data [12], a recent reconstruction of the atomic structure of two PB2 domains [29] has shown that all seven characteristic sites in these domains were located on the protein surface, suggesting their interaction with other viral proteins, or with host factors. Unfortunately, we are currently unable to map these interactions more accurately, either because of insufficient structural information, or because binding regions are identified on only one of the binding proteins. In addition, the available number of influenza A sequences prior to 1950 is insufficient for the statistical identification of co-evolving residues. Even so, our data clearly indicates that internal protein constellations form stable lineages in humans, and natural reassortments of internal proteins do not tend to occur between these lineages. Such lack of reassortments is remarkable given the genetic similarity and overlapping geographical spread of the two lineages, and suggests a very strong interdependency between the elements of the constellation.

The reassortment of the PB1 segment in multiple pandemic events may indicate that stable PB1 mutations are not required for human host adaptation. The paucity of adaptive mutation sites in PB1, when compared to PB2 and PA, suggests it may play a core enzymatic role in the trimeric polymerase complex, while the remaining two subunits are responsible for interactions with host factors. Recent research has proposed a critical role of the PB1 gene in the high virulence of the 1918 pandemic [30], and it is possible that flexibility in replacing this segment is of benefit to the virus at the onset of pandemics. The repeated emergence of the PB1 V336I mutation suggests that it plays an important adaptive role that should be further investigated.

The unusually high density of characteristic sites in the M2 protein may be explained by its physical arrangement in the virion assembly: M2 is a transmembrane protein, thought to interact both with the internal proteins and with the host immune system. The extracellular region (M2e) of this protein was observed to be conserved in humans, and thus proposed as a vaccine candidate [31]. Recently, further studies have claimed that M2e-based vaccines may confer immune protection against zoonotic strains [24]. Our results suggest that the M2 and in particular its M2e domain are prone to developing adaptive mutations. Its conservation in the two human lineages is a poor indicator of its conservation in avian viruses. In view of our incomplete knowledge of avian influenza diversity, claims of universal protection against avian strains should be regarded with caution, especially because of the ease with which reassortments occur in these viruses.

One characteristic mutation observed in the NS1 protein (the introduction of a threonine residue at position 215) affects a Src homology 3 (SH3) motif that is present in many avian isolates. This motif is believed to recruit Crk and CrkL adaptive proteins, and thus modulate signaling pathways that affect the replication ability of the virus [32]. The proline residue required by the SH3 motif was observed in 81% of A2A and 93% of H5N1A isolates. Notably, it was present in the 1918 Spanish influenza isolates and in all but one human-infecting H5N1 viruses. Two other NS1 characteristic sites (positions 81 and 84) are located in a 5-amino acid region (80–84) whose deletion is associated with the second wave of human-infecting H5N1 viruses (see Figure 7).

In spite of their demonstrated flexibility in the composition of their protein constellations, swine viruses have established at least one lineage that has retained several H2H mutations for over three-quarters of a century. Such continuity is in marked contrast with the frequency of reassortment events, which would lead one to expect a number of lineages to have emerged. This suggests that conserved adaptive mutations in “classic swine” are important for swine-to-swine transmission, and essential to the establishment of stable lineages, but not prerequisites for infecting this host. This has important implications for human influenza, since nearly all of these mutations are also conserved in human strains, and have been present since the 1918 Spanish pandemic. The eleven adaptive variants shared by “classical swine” and human signatures may constitute a basic “adaptive suite” for within-species transmission, essential for founding stable lineages. However, this set accounts for only about half of the 1918 H2H mutations, suggesting that major additional changes were required before influenza viruses could spread efficiently among humans.

The pandemic H1N1/09 virus appears to possess H2H mutations similar to those of other swine viruses. Normal “classic” swine viruses possess several such mutations, and it is possible that low-pathogenicity human infections by these strains occur frequently. The avian signature of the PA and PB2 proteins of H1N1/09 suggests suboptimal replication in human hosts, but this might be compensated by the human PB1 recombinant protein. Although the rapid pandemic spread of H1N1/09 may appear inconsistent with suboptimal host adaptation, it is possible that the advantages conferred by the antigenic novelty of its HA and NA proteins were sufficient for the virus to overcome replication disadvantages. As more people develop immunity and its antigenic novelty decreases, H1N1/09 may disappear, or establish itself as a stable human lineage. Either way, the outcome will be extremely informative in assessing the H2H mutations present in this virus' signature.

### Assessment of Avian Influenza Viruses

In our analysis of avian influenza, signatures from H5N1 isolates stood out as the richest in H2H mutations. This result was by no means expected, and it strongly supports the utility of our characteristic site catalogue as an assessment tool. A comparison of 1997 Hong Kong H5N1 signatures against those of contemporary H9N2 and H6N1 isolates from the same geographical region reveals a dynamic interplay between these three subtypes, in which viral segments appear to have been transferred through reassortments (Figure S2 of the Supplementary Materials S1). This observation supports previous studies, which have proposed that the 1997 Hong Kong H5N1 epidemic followed the reassortment of H5N1 and H9N2 viruses [33], and that H6N1 viruses were also involved [34]. Such highly dynamic composition of the avian influenza proteome puts into question the validity of labeling influenza isolates exclusively by their HA and NA subtypes. H5N1 isolates of 1981, 1997 and 2004 clearly present distinct internal protein constellations, and grouping them into a homogeneous set reveals little about their ability to adapt to humans. In addition, an excessive focus on the HA/NA subtype deviates attention from the analysis of co-circulating strains with a potential for reassortment, impairing effective surveillance of the potential for human infectivity and transmissibility. Glycoproteins must be considered as important components of a larger systemic ensemble of adaptations, some of which can only be modeled by new approaches that transcend current subtype definitions.

Remarkably, the two H5N1 waves only share one conserved H2H mutation (M2 I28V), while all other mutations involved in the 1997 waves have been replaced by avian variants. Thus, it appears that H5N1 viruses are not only acquiring, but also losing H2H mutations through reassortments. The lack of stability of adaptive variants is evidenced by the instability of the crucial PB2 E627K mutation, implicated in replication in humans [35] and high virulence of human H5N1 infections [36]. Overall, there is no evidence of a trend of gradual accumulation of H2H mutations in H5N1 viruses. This may indicate that H5N1, in its current form, poses a relatively low pandemic risk. On the other hand, the abundant evidence of reassortments among H5N1 raises the concern that these avian viruses may reassort with a human lineage, combining a human-adapted internal protein constellation with an immunologically novel set of glycoproteins. Such reassortants have been produced under laboratory conditions, using human H3N2 viruses, but have failed to propagate amongst mammalian models [37]. Even if reassortants acquired the ability to circulate efficiently among humans, it is impossible to predict how such adaptation would affect the extreme pathogenicity that has characterized human H5N1 infections: like transmissibility, pathogenicity appears to be systemically determined, and is likely to be affected by the replacement of internal proteins. Since there is no evidence of a link between virulence and host adaptation, our results cannot help make such a prediction.

### Conclusions

In this study, we performed analysis based on mutual information to a dataset of over 40,000 influenza protein sequences, to identify adaptive mutations associated with human-to-human transmissibility. The 68 characteristic sites found by our analysis constitute the most comprehensive catalogue published to date, and a useful tool for assessing the potential for human transmission of influenza viruses. Characteristic sites were found in eight internal proteins, suggesting that adaptation to the human population is complex and may require the orchestration of concurrent mutations in multiple proteins. A remarkable product of this complex adaptive system is the stability of internal protein constellations in the two lineages that circulate amongst humans. These lineages developed most of their repertoire of adaptive mutations gradually over the three decades following the 1918 Spanish influenza pandemic, and have conserved these mutations to the present day. Their internal protein constellations appear so interdependent that the two lineages never reassort with each other or with zoonotic viruses. By contrast, we observed that avian strains are subject to frequent reassortments, which may have helped the accumulation of human adaptive mutations in avian H5N1 prior to the 1997 Hong Kong wave. However, such accumulation has not been stable, and H5N1 adaptations to human hosts are often lost through reassortments. By this measure, the current genetic forms of H5N1 viruses appear to present a limited pandemic risk, except in the event of a reassortment with a human lineage, which is likely to affect the viruses' pathogenicity. H1N1/09 viruses appear similar to existing swine lineages, which multiple H2H mutations that may account for its capability to transmit among humans. Like the 1977 H1N1 pandemic strain, H1N1/09 may have enjoyed rapid spread because of partial serological novelty, but may ultimately be limited by cross-reactive immune memory in a large portion of the population.

The catalogue produced by this study is consistent with the results of previous studies, and our mutual information method appears to be the most powerful and sensitive amongst those proposed thus far. Whilst our catalogue of adaptive sites might contain some false positives, it is clear that this set of positions serves as a useful starting point for verification studies and further research. These studies will include both advanced computational analyses and experimental verification by researchers interested in the epidemiology and evolutionary behavior of influenza viruses, including characterization of molecular mechanisms. Our results show that our understanding of the mechanisms involved in influenza adaptation to humans is incomplete; we propose that a systemic perspective that considers constellations of adaptations should be studied.

## Supporting Information

Supplementary Materials S1 (0.27 MB DOC)
