# Supplementary material for: Complete-Proteome Mapping of Human Influenza A Adaptive Mutations: Implications for Human Transmissibility of Zoonotic Strains
Source: PLoS One. 2010 Feb 3;5(2):e9025. doi: 10.1371/journal.pone.0009025 (PMC2815782; doi:10.1371/journal.pone.0009025)
Supplement: Supplementary Materials S1 — (0.27 MB DOC) [file pone.0009025.s001.doc]

# Supplementary Materials

# Tables

## Table S1 – Catalogue of subtype-specific characteristic sites for the HA protein, subtype H1

Each row represents a site where characteristic variants observed in human-circulating strains were statistically different from those circulating in birds. From left to right, the columns represent: the position in the protein; the residues observed in A2A strains; the residues observed in H2H strains; the conservation of characteristic variants in H2H viruses; the cross-presence of A2A characteristic variants in H2H viruses.

## Table S2 – Catalogue of subtype-specific characteristic sites for the HA protein, subtype H2

Each row represents a site where characteristic variants observed in human-circulating strains were statistically different from those circulating in birds. From left to right, the columns represent: the position in the protein; the residues observed in A2A strains; the residues observed in H2H strains; the conservation of characteristic variants in H2H viruses; the cross-presence of A2A characteristic variants in H2H viruses.

## Table S3 – Catalogue of subtype-specific characteristic sites for the HA protein, subtype H3

Each row represents a site where characteristic variants observed in human-circulating strains were statistically different from those circulating in birds. From left to right, the columns represent: the position in the protein; the residues observed in A2A strains; the residues observed in H2H strains; the conservation of characteristic variants in H2H viruses; the cross-presence of A2A characteristic variants in H2H viruses.

## Table S4 – Catalogue of subtype-specific characteristic sites for the NA protein, subtype N1

Each row represents a site where characteristic variants observed in human-circulating strains were statistically different from those circulating in birds. From left to right, the columns represent: the position in the protein; the residues observed in A2A strains; the residues observed in H2H strains; the conservation of characteristic variants in H2H viruses; the cross-presence of A2A characteristic variants in H2H viruses.

## Table S5 – Catalogue of subtype-specific characteristic sites for the NA protein, subtype N2

Each row represents a site where characteristic variants observed in human-circulating strains were statistically different from those circulating in birds. From left to right, the columns represent: the position in the protein; the residues observed in A2A strains; the residues observed in H2H strains; the conservation of characteristic variants in H2H viruses; the cross-presence of A2A characteristic variants in H2H viruses.

Table S1

| **H1** | | | | |
| --- | --- | --- | --- | --- |
| Position | CV | | H2H | |
|  | A2A | H2H | Cons. | X-pres |
| 2 | E | K | 98.87% | 0.65% |
| 6 | F | L | 98.42% | 0.16% |
| 9 | F | L | 99.21% | 0.79% |
| 14 | V | A | 99.84% | 0.00% |
| 15 | LA | T | 98.60% | 1.25% |
| 16 | K | YD | 98.13% | 0.16% |
| 22 | V | I | 99.22% | 0.65% |
| 60 | NS | DL | 88.18% | 2.90% |
| 62 | N | SK | 94.80% | 4.58% |
| 71 | DK | N | 94.42% | 5.52% |
| 78 | L | I | 96.97% | 1.94% |
| 85 | D | E | 98.42% | 1.45% |
| 88 | L | IF | 95.27% | 4.37% |
| 89 | DT | N | 76.77% | 0.51% |
| 90 | A | K | 94.54% | 1.58% |
| 91 | NS | EK | 94.54% | 1.46% |
| 97 | I | VA | 99.39% | 0.61% |
| 100 | S | P | 98.30% | 1.46% |
| 111 | E | Y | 92.60% | 0.12% |
| 113 | I | A | 97.09% | 2.31% |
| 130 | K | R | 99.27% | 0.73% |
| 137 | A | EG | 96.48% | 0.61% |
| 144 | ED | TN | 98.30% | 1.33% |
| 145 | T | V | 94.18% | 4.00% |
| 154 | Y | H | 98.67% | 1.09% |
| 155 | SL | NK | 94.91% | 0.24% |
| 157 | AG | KER | 98.67% | 1.33% |
| 167 | I | L | 99.52% | 0.12% |
| 169 | K | GE | 96.48% | 2.79% |
| 171 | G | N | 96.12% | 1.45% |
| 172 | TND | G | 98.30% | 1.09% |
| 176 | KE | N | 94.91% | 2.91% |
| 182 | TI | VA | 99.64% | 0.36% |
| 200 | TS | N | 98.19% | 1.81% |
| 201 | TVA | IM | 97.66% | 0.12% |
| 202 | SND | GER | 96.31% | 0.37% |
| 203 | E | DN | 95.90% | 1.74% |
| 205 | Q | RK | 96.93% | 2.95% |
| 206 | S | A | 79.58% | 1.48% |
| 209 | Q | HR | 94.83% | 2.58% |
| 210 | N | TK | 97.29% | 2.71% |
| 211 | ATV | E | 97.05% | 1.35% |
| 212 | D | N | 98.03% | 1.23% |
| 218 | G | VL | 97.83% | 1.53% |
| 221 | K | HN | 98.34% | 1.28% |
| 232 | A | KE | 97.96% | 1.28% |
| 243 | M | I | 95.66% | 4.34% |
| 251 | D | E | 99.23% | 0.13% |
| 252 | Q | P | 99.62% | 0.13% |
| 257 | VNT | LI | 95.37% | 4.32% |
| 261 | T | N | 97.09% | 2.41% |
| 274 | NK | S | 96.33% | 3.54% |
| 275 | K | R | 99.11% | 0.76% |
| 277 | S | F | 95.95% | 1.39% |
| 278 | D | G | 98.23% | 0.00% |
| 285 | D | N | 98.35% | 1.01% |
| 288 | VI | M | 98.35% | 1.27% |
| 289 | H | DG | 92.03% | 2.91% |
| 290 | N | E | 97.34% | 0.13% |
| 299 | HYKR | Q | 98.23% | 1.39% |
| 302 | L | I | 99.87% | 0.13% |
| 314 | I | V | 98.86% | 0.63% |
| 324 | K | R | 98.57% | 1.43% |
| 337 | V | I | 98.30% | 1.57% |
| 415 | N | K | 96.21% | 2.04% |
| 420 | I | M | 97.96% | 2.04% |
| 434 | V | I | 98.83% | 1.17% |
| 459 | R | K | 99.71% | 0.30% |
| 470 | R | K | 97.05% | 2.95% |
| 515 | E | KR | 98.52% | 1.48% |

Table S2

| **H2** | | | | |
| --- | --- | --- | --- | --- |
| Position | CV | | H2H | |
|  | A2A | H2H | Cons. | X-pres |
| 60 | S | LR | 95.63% | 0.12% |
| 62 | NR | K | 99.27% | 0.61% |
| 71 | K | N | 88.97% | 10.91% |
| 89 | T | S | 96.84% | 1.34% |
| 104 | NH | YD | 97.14% | 2.86% |
| 126 | T | K | 100.00% | 0.00% |
| 136 | R | K | 100.00% | 0.00% |
| 139 | Q | R | 92.86% | 0.00% |
| 252 | TI | MV | 98.57% | 0.00% |
| 257 | T | I | 98.21% | 1.15% |
| 311 | I | V | 100.00% | 0.00% |
| 327 | R | K | 100.00% | 0.00% |
| 388 | IV | F | 100.00% | 0.00% |
| 473 | A | V | 100.00% | 0.00% |
| 523 | N | S | 100.00% | 0.00% |

Table S3

| **H3** | | | | |
| --- | --- | --- | --- | --- |
| Position | CV | | H2H | |
|  | A2A | H2H | Cons. | X-pres |
| 13 | AIT | V | 97.48% | 2.17% |
| 15 | SG | A | 94.96% | 4.28% |
| 38 | D | N | 97.82% | 0.92% |
| 57 | K | RG | 94.75% | 4.07% |
| 60 | N | D | 95.73% | 3.67% |
| 62 | N | S | 95.79% | 3.60% |
| 70 | R | EK | 96.40% | 0.10% |
| 71 | D | N | 98.13% | 1.84% |
| 86 | V | G | 96.73% | 0.97% |
| 89 | D | N | 99.67% | 0.30% |
| 92 | T | EK | 97.73% | 2.20% |
| 101 | NS | K | 91.50% | 0.17% |
| 135 | TQ | NK | 98.38% | 1.23% |
| 139 | T | ND | 97.80% | 2.10% |
| 156 | P | S | 96.31% | 3.56% |
| 157 | AT | NVID | 98.58% | 0.29% |
| 159 | G | S | 96.50% | 3.43% |
| 170 | G | KE | 94.49% | 3.11% |
| 173 | AT | K | 94.26% | 3.66% |
| 186 | N | K | 92.59% | 4.75% |
| 201 | N | D | 94.60% | 2.85% |
| 202 | Q | SN | 85.32% | 2.43% |
| 220 | R | K | 97.87% | 1.81% |
| 226 | I | V | 96.06% | 3.85% |
| 239 | Q | VLI | 96.22% | 3.46% |
| 241 | G | S | 99.90% | 0.00% |
| 255 | V | I | 98.57% | 1.23% |
| 257 | V | L | 96.63% | 3.08% |
| 261 | N | T | 94.55% | 4.51% |
| 273 | M | I | 95.82% | 3.60% |
| 292 | IV | NS | 97.27% | 2.34% |
| 321 | K | R | 95.05% | 4.92% |
| 345 | L | I | 96.72% | 3.11% |
| 493 | E | G | 95.92% | 4.01% |
| 504 | I | V | 99.86% | 0.07% |
| 556 | R | K | 98.45% | 1.55% |

Table S4

| **N1** | | | | |
| --- | --- | --- | --- | --- |
| Position | CV | | H2H | |
|  | A2A | H2H | Cons. | X-pres |
| 47 | E | G | 97.71% | 0.57% |
| 48 | TPK | IV | 95.14% | 3.43% |
| 52 | S | R | 92.86% | 4.00% |
| 59 | N | S | 93.14% | 4.00% |
| 90 | G | S | 94.48% | 4.36% |
| 142 | T | A | 95.94% | 2.03% |
| 148 | V | LI | 94.19% | 4.65% |
| 173 | IV | M | 88.54% | 0.57% |
| 174 | S | G | 98.28% | 0.57% |
| 199 | D | E | 96.86% | 1.43% |
| 206 | NG | K | 97.43% | 1.43% |
| 207 | ND | RQKE | 97.71% | 0.86% |
| 217 | A | V | 96.57% | 1.43% |
| 235 | Q | AP | 94.86% | 2.57% |
| 249 | V | T | 93.12% | 0.29% |
| 252 | V | I | 95.13% | 3.44% |
| 270 | AS | T | 97.42% | 0.86% |
| 272 | E | T | 92.26% | 0.29% |
| 296 | E | D | 98.57% | 0.29% |
| 314 | ND | KR | 97.41% | 1.15% |
| 317 | T | EK | 97.13% | 1.44% |
| 324 | S | TNY | 96.84% | 1.15% |
| 325 | SL | VA | 97.70% | 1.15% |
| 326 | NS | D | 97.70% | 1.15% |
| 329 | YF | DN | 98.85% | 0.00% |
| 336 | F | Y | 97.70% | 1.15% |
| 350 | TI | N | 95.94% | 1.45% |
| 354 | S | KQ | 96.81% | 0.87% |
| 371 | S | D | 66.67% | 1.47% |
| 439 | GS | ND | 98.23% | 1.18% |

Table S5

| **N2** | | | | |
| --- | --- | --- | --- | --- |
| Position | CV | | H2H | |
|  | A2A | H2H | Cons. | X-pres |
| 40 | N | Y | 81.77% | 0.00% |
| 44 | IN | S | 98.10% | 0.00% |
| 46 | SA | P | 90.05% | 9.14% |
| 53 | VTI | M | 98.73% | 0.75% |
| 78 | Q | NKD | 94.88% | 4.95% |
| 132 | G | D | 97.20% | 1.14% |
| 185 | R | E | 55.95% | 1.23% |
| 196 | MI | R | 99.50% | 0.06% |
| 244 | RK | E | 97.48% | 2.52% |
| 272 | END | G | 97.37% | 2.63% |
| 289 | IL | V | 96.79% | 3.15% |
| 293 | AT | KE | 99.94% | 0.00% |
| 329 | P | HQ | 97.44% | 2.33% |
| 360 | R | K | 99.77% | 0.06% |
| 366 | TAI | S | 99.60% | 0.00% |
| 368 | A | P | 98.63% | 0.63% |
| 381 | N | R | 86.07% | 1.26% |
| 382 | N | G | 82.46% | 2.52% |
| 384 | WSL | R | 96.10% | 2.35% |
| 416 | P | KEN | 97.42% | 0.97% |

# Figures

## Figure S1 – Adaptation signatures of selected avian influenza A proteomes containing multiple H2H mutations.

Selected signatures of avian proteomes that were found to contain 4 or more H2H mutations are shown. For conciseness, a number of similar signatures were removed from this set. Subtype, year and country of isolation, host (for avian viruses isolated in humans) and isolate name are shown in the first column, while the second column shows the number of H2H mutations. The remaining columns show the signature residues, using the same colouring scheme as in Figure 6 of the main text. Asian strains of subtypes H5N1, H9N2 and H6N1 are placed above the red horizontal line, while signatures from other subtypes, mostly sampled from gulls and shorebirds, are shown below the line.

## Figure S2 – Adaptation signatures of recent swine influenza A proteomes with novel signatures.

This table shows the signatures of three recent isolates from Korea, exhibiting novel signatures and probable reassortments. A “classical swine” influenza signature is provided (above the red line) for comparison.
